# Supplementary material for: General medical publications during COVID-19 show increased dissemination despite lower validation
Source: PLoS One. 2021 Feb 2;16(2):e0246427. doi: 10.1371/journal.pone.0246427 (PMC7853485; doi:10.1371/journal.pone.0246427)
Supplement: S1 Table — (DOCX) [file pone.0246427.s002.docx]

S1 Table – Publication characteristics and impact by journal

| **,** | **JAMA** | | | **Lancet** | | | **Nature Medicine** | | | **NEJM** | | |
| --- | --- | --- | --- | --- | --- | --- | --- | --- | --- | --- | --- | --- |
|  | **2019** | **Non-COVID** | **COVID** | **2019** | **Non-COVID** | **COVID** | **2019** | **Non-COVID** | **COVID** | **2019** | **Non-COVID** | **COVID** |
| **Articles (n)** | 38 | 40 | 21 | 53 | 28 | 17 | 29 | 29 | 12 | 52 | 57 | 26 |
| **Article type, No. (%)** |  | | |  | | |  | | |  | | |
| Meta-analysis | 1 (3%) | 1 (3%) | 0 (0%) | 3 (6%) | 2 (7%) | 0 (0%) | 2 (7%) | 1 (3%) | 0 (0%) | 0 (0%) | 0 (0%) | 0 (0%) |
| Systematic review | 1 (3%) | 5 (13%) | 1 (5%) | 3 (6%) | 1 (4%) | 1 (6%) | 0 (0%) | 0 (0%) | 0 (0%) | 0 (0%) | 0 (0%) | 0 (0%) |
| Narrative review | 2 (5%) | 3 (8%) | 0 (0%) | 9 (17%) | 6 (21%) | 3 (18%) | 1 (3%) | 1 (3%) | 0 (0%) | 5 (10%) | 6 (11%) | 1 (4%) |
| RCT | 15 (39%) | 7 (18%) | 0 (0%) | 25 (47%) | 15 (54%) | 0 (0%) | 0 (0%) | 4 (14%) | 0 (0%) | 23 (44%) | 24 (42%) | 1 (4%) |
| Cohort / prospective | 10 (26%) | 14 (35%) | 12 (57%) | 7 (13%) | 0 (0%) | 2 (12%) | 6 (21%) | 5 (17%) | 1 (8%) | 7 (13%) | 10 (18%) | 9 (35%) |
| Case-control | 0 (0%) | 2 (5%) | 0 (0%) | 1 (2%) | 0 (0%) | 0 (0%) | 1 (3%) | 1 (3%) | 2 (17%) | 1 (2%) | 1 (2%) | 0 (0%) |
| Case report or series | 1 (3%) | 1 (3%) | 8 (38%) | 3 (6%) | 3 (11%) | 7 (41%) | 0 (0%) | 0 (0%) | 3 (25%) | 10 (19%) | 13 (23%) | 13 (50%) |
| Basic biomedical research / preclinical | 0 (0%) | 0 (0%) | 0 (0%) | 0 (0%) | 0 (0%) | 0 (0%) | 17 (59%) | 17 (59%) | 3 (25%) | 1 (2%) | 1 (2%) | 2 (8%) |
| Other | 8 (21%) | 7 (18%) | 0 (0%) | 2 (4%) | 1 (4%) | 4 (24%) | 2 (7%) | 0 (0%) | 3 (25%) | 5 (10%) | 2 (4%) | 0 (0%) |
| **Study characteristics, No. (%)** |  | | |  | | |  | | |  | | |
| Registered trial | 17 (59) | 8 (20) | 0 (0) | 25 (50) | 16 (57) | 0 (0) | 3 (11) | 7 (25) | 0 (0) | 30 (59) | 23 (40) | 0 (0) |
| Industry funding | 9 (25) | 6 (15) | 0 (0) | 6 (11) | 11 (39) | 0 (0) | 4 (14) | 9 (31) | 0 (0) | 18 (35) | 22 (39) | 2 (8) |
| **Publication characteristics** |  | | |  | | |  | | |  | | |
| Author number, median (IQR) | 9.5 (12.25) | 7 (8.25) | 7 (2.5) | 15 (16.5) | 16 (23) | 7 (9) | 21 (16.5) | 16 (24) | 13.5 (7) | 15 (16.75) | 13 (15.5) | 17 (17.75) |
| Author affiliations, median (IQR) | 5 (12.25) | 4 (6.5) | 3 (2) | 8 (16.5) | 12 (16) | 5 (5.5) | 10 (10) | 9 (13.5) | 4 (4.75) | 8 (13.75) | 7 (13.5) | 4.5 (6) |
| Female corresponding or first author **^a^**, No. (%) | 14 (36.8) | 13 (32.5) | 8 (42) | 14 (28) | 11 (39.3) | 5 (29.4) | 13 (50) | 15 (53.1) | 3 (27.3) | 18 (37.5) | 17 (30.4) | 8 (33.3) |
| Time to publication (days), mean (SD) | N/A | N/A | N/A | N/A | N/A | N/A | 305.3 (124.2) | 288.3 (99.7) | 35.1 (4.6) | N/A | N/A | N/A |
| Word count, mean (SD) | 3347 (1704) | 3044 (1541) | 1399 (1404) | 5797 (3632) | 5216 (2207) | 2694 (1806) | 4412 (3082) | 7399 (2426) | 2385 (968) | 3167 (836) | 3034 (1096) | 1283 (1344) |
| References, mean (SD) | 31 (26) | 33 (25) | 13 (21) | 59 (67) | 50 (45) | 31 (35) | 51 (20) | 53 (17) | 23 (15) | 29 (18) | 30 (18) | 11 (15) |
| **Publication impact ^b^, median (IQR)** |  | | |  | | |  | | |  | | |
| Reads **^c^** | 12765 (13234) | 6918 (12237) | 137588 (352726) | -- | -- | -- | 12000 (11328) | 4625 (2737) | 304000 (298500) | 24966 (25097) | 17640 (14563) | 255705 (473907) |
| Tweets | 151 (280.25) | 69 (189) | 1159 (2342) | 156 (193.5) | 61.5 (89) | 674.5 (6433.7) | 148 (269) | 102 (124) | 4087 (3847.5) | 240 (302.8) | 82 (176.5) | 1192 (5758.2) |
| Times cited | 14.5 (21.25) | 1 (4) | 25 (120.5) | 22 (32) | 2 (3) | 22 (46) | 42 (35) | 2 (4.5) | 78 (130.05) | 27.5 (43.25) | 2 (7.5) | 100 (171) |

Abbreviations: COVID, Coronavirus Disease; *JAMA*, Journal of the American Medical Association; *NEJM*, New England Journal of Medicine; RCT, Randomized Controlled Trial

**^a^** Articles in which study characteristic was not reported or in which gender of author was unknown were excluded from calculation of the proportion.

**^b^** Reads, tweets and times cited are reported as absolute numbers and are not normalized to their time since publication.

**^c^** *The Lancet* does not include article reads as part of their Altmetrics and excluded here.
